# Nanofibrous Material-Reinforced Printable Ink for Enhanced Cell Proliferation and Tissue Regeneration

**DOI:** 10.3390/bioengineering11040363

**Published:** 2024-04-11

**Authors:** Iruthayapandi Selestin Raja, Bongju Kim, Dong-Wook Han

**Affiliations:** 1 Institute of Nano-Bio Convergence, Pusan National University, Busan 46241, Republic of Korea; 2 Dental Life Science Research Institute, Seoul National University Dental Hospital, Seoul 03080, Republic of Korea; bjkim016@gmail.com; 3 Department of Cogno-Mechatronics Engineering, College of Nanoscience & Nanotechnology, Pusan National University, Busan 46241, Republic of Korea

**Keywords:** printable ink, tissue regeneration, hydrogel, nanofiber-reinforced ink

## Abstract

The three-dimensional (3D) printing of biomaterials, cells, and bioactive components, including growth factors, has gained interest among researchers in the field of tissue engineering (TE) with the aim of developing many scaffolds to sustain size, shape fidelity, and structure and retain viable cells inside a network. The biocompatible hydrogel employed in 3D printing should be soft enough to accommodate cell survival. At the same time, the gel should be mechanically strong to avoid the leakage of cells into the surrounding medium. Considering these basic criteria, researchers have developed nanocomposite-based printable inks with suitable mechanical and electroconductive properties. These nanomaterials, including carbon family nanomaterials, transition metal dichalcogenides, and polymeric nanoparticles, act as nanofillers and dissipate stress across polymeric networks through their electroactive interactions. Nanofiber-reinforced printable ink is one kind of nanocomposite-based ink that comprises dispersed nanofiber components in a hydrogel matrix. In this current review, we compile various TE applications of nanofiber-reinforced printable ink and describe the 3D-printing parameters, classification, and impact of cross-linkage. Furthermore, we discuss the challenges and future perspectives in this field.

## 1. Introduction

**Figure 1:**
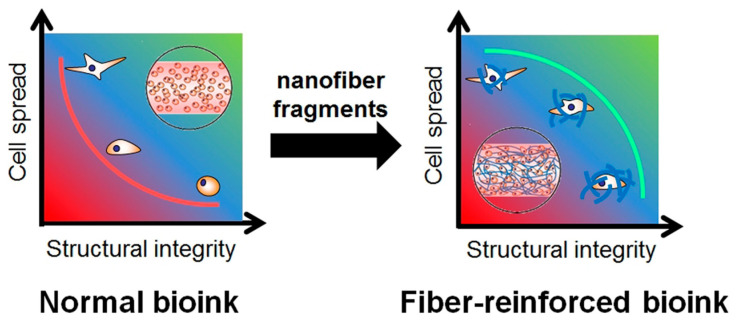
Differences in relationship between structural integrity and cell proliferation in normal and nanofiber-reinforced printable inks. Data reproduced from Ref. [6]. Copyright Elsevier 2020.

Tissue engineering (TE) is a promising strategy for healing severely injured tissues and organs by promoting cellular growth and avoiding contamination. TE involves the contribution of cells, scaffolds, and/or growth factors. Among these, scaffolds exhibit a significant role in providing a suitable microenvironment for cell attachment and proliferation by imitating the extracellular matrix (ECM) [1]. Fabricated biomaterials are mostly in the form of hydrogels, sponges, thin films, and nanofiber mats that imitate the ECM [2,3,4]. Several techniques have been employed to fabricate scaffolds with the appropriate physicochemical and biological properties. Here, 3D-printing technology is gaining interest among researchers in TE-relevant fields for its unique ability to precisely fabricate tissue constructs with the desired shape and size [5]. Hydrogels can be extruded by this technique with high reproducibility. There are certain conditions required for the hydrogels employed in bioprinting. The hydrogel should be soft, mechanically tolerable, and biocompatible, providing support for cell migration and cell–material interactions. A mechanically weak hydrogel loses shape fidelity and stability during extrusion and may cause the leakage of cells into the medium. Meanwhile, a tough hydrogel imposes constraints on the cells residing inside the 3D structure, eventually causing their death within the scaffold [6]. Considering these points, researchers have focused on developing various hybrid hydrogel constructs by incorporating different types of additives that improve physicochemical properties while providing favorable microenvironmental cues to the cells. The reinforcing materials used to develop nanocomposite-based printable inks can be nanoparticles, nanofibers, nanoclays, etc., which require a compromise between printability and biocompatibility [7,8,9,10]. Particularly, nanofibrous-material-reinforced hydrogels have been reported to act as supporting platforms for structural integrity and cell spread and imitate natural ECM-containing soft tissues (Figure 1). In this review, we compiled tissue engineering applications of printable inks composed of nanofibers and their fragments.

## 2. Three-Dimensional Bioprinting

Hydrogels are 3D crosslinked networks of hydrophilic polymers that can swell by absorbing about 70–99% of their content as water. The entrapment of water molecules into hydrogels makes them porous and permeable [11]. Owing to these properties, hydrogels can provide initial support for cell growth and remodel their surroundings when the cells proliferate. Hydrogels can be prepared from natural biomacromolecules such as collagen, hyaluronic acid, elastin, keratin, etc., and synthetic polymers like polyvinyl alcohol, polylactic acid, and polyethylene glycol [12,13,14,15]. They are classified into homopolymeric, copolymeric, and multipolymer interpenetrating polymeric hydrogels according to their polymeric composition. The presence or absence of electrical charges on the polymeric chains categorizes them as nonionic, neutral, amphoteric, and zwitterionic hydrogels [16,17]. A hydrogel employed for 3D bioprinting requires a specific set of conditions for cellular functions: an aqueous environment, appropriate pH and osmolarity, diffusion of oxygen and nutrients, and the presence of vital vitamins and minerals. For certain cell types, like skin fibroblasts, the matrix should contain cell attachment sites with the ability to proliferate. When the printed materials degrade or fragment over time, the released components should be nontoxic and not interrupt new tissue formation [10].

**Figure 2:**
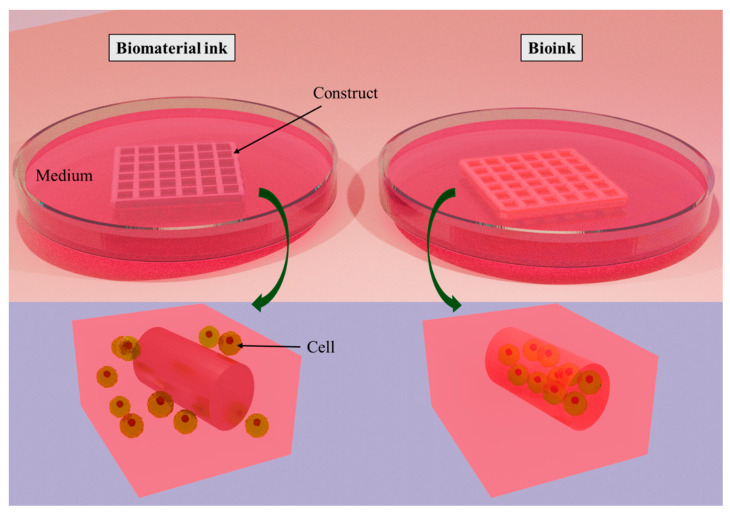
Classification of printable ink: biomaterial ink and bioink. The difference between them is that cells are seeded on biomaterial ink after printing, whereas cells are encapsulated into bioink during bioprinting. Created with docs.blender.org.

Printable ink can be classified into two categories: biomaterial ink and bioink (Figure 2). Biomaterial ink does not contain cells in its formulations and hence can be sterilized and post-treated (crosslinking, washing, etc.) a long time before cell seeding. The cells can grow abundantly on the scaffold’s surface and can migrate to its core part to some extent. In the case of cell-laden bioink, cells are mixed with polymeric components and other bioactive components before bioprinting; hence, care must be taken in terms of biocompatibility and post-crosslinking time to ensure cell survival [18]. Bioinks can be either cell-scaffold-based or scaffold-free cell-based formulations, depending on the development of the target tissue-like structures [19].

**Figure 3:**
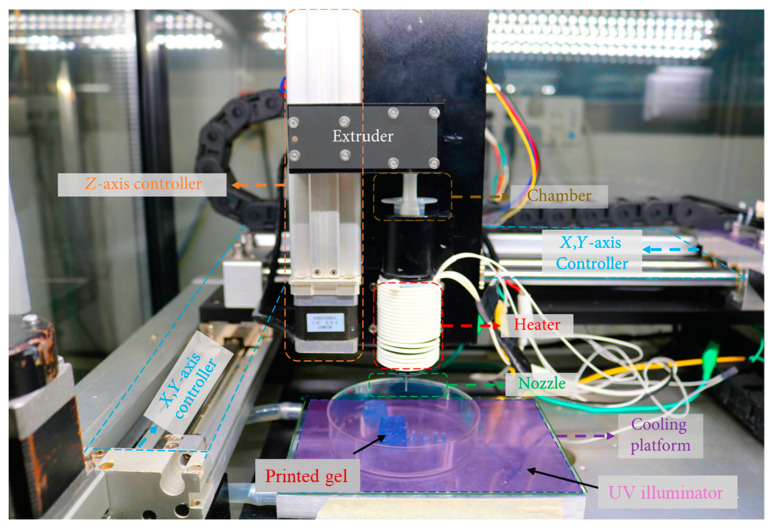
A typical 3D extrusion based bioprinter. Data reproduced from Ref. [21]. Copyright Hindawi 2020.

The bioprinting process depends on several specified parameters based on geometry, process, and structure. The geometry-based parameters are nozzle size and filament size. Bed temperature, melting temperature, and printing speed are the process-based parameters. Layer thickness, infill density, raster angle, and raster gap are considered structural-based parameters [20]. These physical parameters play a key role in determining the precision and properties of any fabricated printable ink. A typical 3D extrusion-based bioprinter is shown in Figure 3.

## 3. Nanocomposite-Reinforced Printable Ink

The important aspects of a printable ink are its structural, mechanical, and biochemical properties during reinforcement approaches to enhance TE. The well-known reinforcement approaches are polymer functionalization [22,23], supramolecular networks [24], nanocomposite-based interactions [25], ionic–covalent entanglement [26,27], and coprinting [28,29]. These approaches strengthen crosslinks, dissipate mechanical energy, and homogenize the stress distribution in a hybrid hydrogel. Methacrylate functionalization is a popular polymer functionalization method in which a polymeric backbone is chemically conjugated with methacrylic anhydride. In the presence of a photoinitiator, the functional methacrylate groups can be photocrosslinked by exposure to UV light [30]. Ionic crosslinkers, including multivalent cations of calcium, ferric, zinc, and strontium, have a significant influence on a material’s printability. In particular, water-soluble calcium ions have been widely used for crosslinking alginate-based inks [31]. The click reaction is highly selective and develops hydrogels with elastic moduli equivalent to those of methacryloyl hydrogels. The resulting hydrogels possess a homogeneous network, which improves fracture toughness and extensibility [32]. For instance, the thiol-ene click reaction conjugates the thiol and alkene groups of polymeric chains, which has gained interest in functionalizing various biomaterials in TE [33]. Supramolecular hydrogels are commonly used as self-healing inks, which are created by the self-assembly of short polymeric chains through noncovalent interactions. Supramolecular networks have viscoelastic properties with an alteration in shear stress. Owing to the reversibility of physical bonds, supramolecular hydrogels have received interest from printable ink researchers [34].

**Figure 4:**
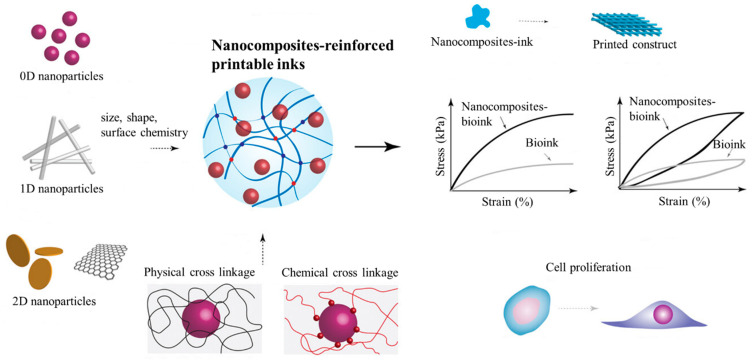
Nanocomposite-reinforced printable ink. Nanomaterials with different dimensions, sizes, shapes, and surface chemistries crosslink bioinks through various physical and chemical interactions. Due to reinforcement, the resulting hybrid hydrogel shows improved printability, mechanical strength, and rheological properties. As the functional nanomaterials disperse the stress across the internal structure, the matrix augments cell attachment and proliferation. Data reproduced from Ref. [10]. Copyright John Wiley and Sons 2019.

The impact of nanomaterials is high in biomedical research due to their large surface area, tunable size and shape, and functionality even at low concentrations [35]. Carbon family nanomaterials, transition metal dichalcogenides, nanoclays, and polymeric nanoparticles have been broadly used as additives to crosslink polymeric hydrogels [36,37,38,39]. Apart from crosslinking, the incorporation of functional nanomaterials into printable inks has been reported to enhance their printability, mechanical properties, electrical conductivity, stimuli sensitivity, and cell–material interactions. Nanocomposite-reinforced printable ink allows the dispersion of stress across the polymeric network, improves stiffness and toughness, and avoids large-scale crack propagation (Figure 4) [40,41]. Printable inks reinforced with nanofibers are some of the most efficient and biocompatible nanocomposite-reinforced inks, which will be studied in the subsequent section, highlighting their importance and biomedical applications in TE.

## 4. Nanofiber-Reinforced 3D-Printable Ink

Hydrogels contain substantial amounts of water and exhibit hydrophilic properties. However, hydrogel matrices are not suitable for adherent cells, which strictly require a base material to attach to inside the matrix to promote proliferation. For instance, Ko YG et al. [6] observed the morphology and cytoskeleton of stained fibroblasts in gelatin methacryloyl (GelMA) hydrogel by fluorescence microscopy; the cells remained in their original spherical shape after 3 days of culturing. Meanwhile, the cells encapsulated in PLGA-nanofiber-containing GelMA demonstrated a partial stretch of F-actins inside the matrix. Adherent cells, in general, attach and spread on a two-dimensional surface in 3 days before the mature proliferation process. But, a hydrogel 3D matrix does not easily accommodate cell survival by responding to biochemical cues generated from adherent cells and hence fails to spread. When a nanomaterial like nanofibers is homogenously dispersed into a hydrogel matrix, it creates an appropriate microenvironment, facilitating cell–cell and cell–matrix communication [42]. Fiber length and density play a pivotal role in determining cell spread in a nanofibrous hydrogel matrix. Recently, Matera DL et al. [43] demonstrated a fiber-density-dependent increase in cell spreading in fibrous hydrogel composites compared to that in hydrogels lacking fibers. They discovered that fiber reinforcement influenced cell shape and fibroblast activation independent of the bulk material. They revealed that increasing the fiber density at a constant elastic modulus of the bulk hydrogel controlled cellular mechanosensing in a 3D environment with increased levels of Yes-associated protein, a mechanosensitive transcription factor. While attempting to establish the relationship between fiber density and myofibroblast differentiation, the same research group reported that myofibroblast spreading in the 3D hydrogel was positively related to the matrix fibers but inversely connected with hydrogel stiffness [44]. Hiraki HL et al. [45] reported that different cell migration modes of MCF10A epithelial cells, including single mesenchymal cells, amoeboids, strands, and clusters, could be tuned by orthogonally altering bulk hydrogel mechanics and fiber density. From these observations, we can understand the importance of reinforcing 3D-printable ink using nanofiber. The in vitro tissue engineering applications of various nanofiber-reinforced printable inks are listed in Table 1. The specifications of the printed constructs, crosslinking methods, and the cells employed are also provided.

### 4.1. Cellulose-Nanofiber-Reinforced Ink

**Figure 5:**
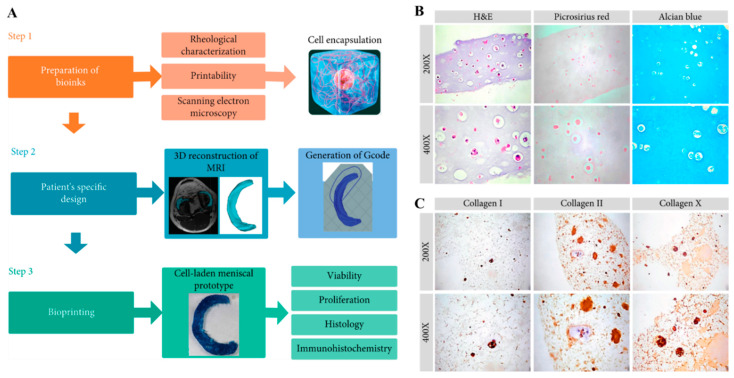
(**A**) Schematic explanation of the preparation of CNF-modified gelatin-alginate bioinks and bioprinting of a cell-laden meniscal prototype based on the patient’s specific design (MRI data). (**B**) Histological results (H&E, picrosirius red, and alcian blue) of bioprinted structures after 14 days of incubation with rabbit fibrochondrocytes. (**C**) Immunohistochemical results of stained collagen I, II, and X in the samples. Data reproduced from Ref. [21]. Copyright Hindawi 2020.

Cellulose nanofibers (CNFs) are compact with an oriented microstructure and good mechanical strength. They are the main constituents of biomass. CNFs are relatively inexpensive, plentiful, and versatile biomaterials that can be employed in a wide variety of tissue engineering applications. CNFs can be divided into three main types, i.e., nanocrystalline cellulose, microfibrillated cellulose, and bacterial cellulose, depending on the source of availability and preparation conditions [55]. The incorporation of CNFs into printable hydrogels ensured shape fidelity and resolution [56]. Viscoelastic bioinks comprising quince seed mucilage and cellulose nanofibrils (QSM/CNF) were fabricated into 3D grid structures through direct ink writing by Baniasadi H et al. [46]. The freeze-dried QSM/CNF inks possessed suitable porosity, mechanical properties, and water uptake capacity due to the presence of CNFs. The highest CNF content endowed the inks with a compressive modulus and an elastic modulus of 32 ± 1 kPa and 64 ± 2 kPa, respectively, which are perfectly fit for soft tissues. The cell-laden bioinks showed cell viability greater than 90% and supported improved cell attachment and proliferation. In similar research [21], CNF-modified gelatin-alginate thermal-responsive bioinks ensured superior-accuracy bioprinting of specific-designed meniscus prototypes than their non-CNF-comprising counterparts. Biological studies revealed that fibrochondrocyte-laden CNF-modified bioink upheld long-term cellular viability, appropriate metabolic activity, and adequate ECM accumulation. Owing to the improved rheological properties, the bioprinted 3D meniscus structure minimized the mismatch with the native knee joint tissues, proving its suitability for repairing meniscal injury (Figure 5).

**Figure 6:**
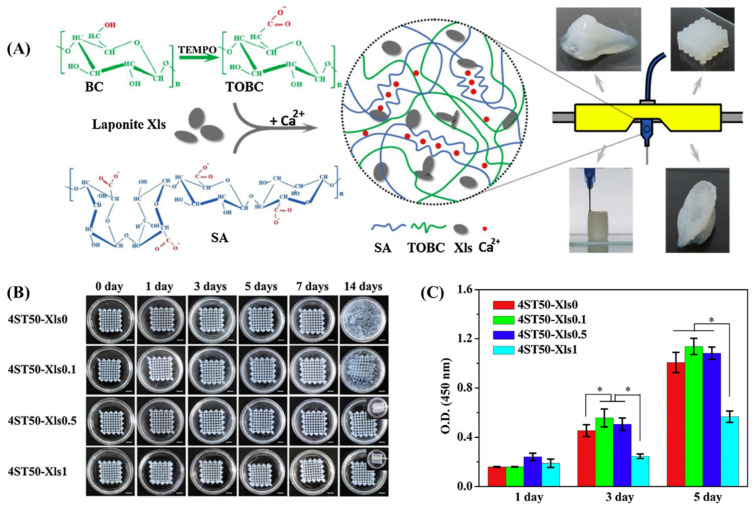
(**A**) Schematic illustration of the fabrication of printed hydrogel constructs from a composite of laponite nanoclay (Xls)-mixed TOBC. BC: bacterial cellulose; TEMPO: 2,6,6-tetramethylpiperidine-1-oxyl radical; TOBC: TEMPO-oxidized bacterial cellulose; SA: sodium alginate. (**B**) The digital images of the printed hydrogel constructs in PBS solution for 14 days with a scale bar of 5 mm. (**C**) The cell proliferation of L929 on the surface of printed constructs on days 1, 3, and 5 post-incubation. A statistically significant difference between the samples was denoted by * *p* < 0.05. Data reproduced from Ref. [48]. Copyright Elsevier 2020.

The bacterial cellulose (BC) produced by *Gluconacetobacter xylinus* is a crystalline nanofiber with a high aspect ratio [55,57]. They have higher hydrophilicity and better mechanical properties than cellulose obtained from other sources. BC nanofibers are morphologically similar to collagen fibrils and have the potential to facilitate cell fate processes [58,59,60]. These nanofibers exhibit an interconnected 3D structure owing to their numerous hydrogen bonds. Because of this feature, BC has been less applied as an additive in composites in tissue engineering. Recently, a 2,6,6-tetramethylpiperidine-1-oxyl radical (TEMPO)-oxidation reaction was established to alter cellulose’s surface and disperse it effectively under ambient conditions. TEMPO-oxidized bacterial cellulose (TOBC) has been extensively used as a promising biomaterial in printable hydrogels for versatile tissue engineering applications [61]. The fibrillated TOBC–sodium alginate composite was crosslinked in the presence of a Ca^2+^ solution to form printable hydrogel composites. TOBC enhanced the chemical, mechanical, and structural stability of the composites and enabled them to act as a template to improve cell viability after cell encapsulation. The TOBC nanofibers surrounding the SA beads induced the formation of cell clusters for the rapid proliferation of viable cells [47]. In another similar work [48], a printable ink of TOBC, sodium alginate, and laponite nanoclay hydrogel composite was reported to exhibit structural stability and achieve the prolonged release of protein for tissue regeneration applications (Figure 6). The hybrid bioink maintained cell viability during the culture period; notably, the cellular morphology was spindle-shaped due to the presence of nanoclay and nanofiber, which assisted in cell–matrix interactions. Furthermore, the bioink, when it had a suitable content of additives, demonstrated a larger cell spread.

TEMPO-oxidized cellulose nanofibers were combined with pectin to develop a multicomponent, L929 fibroblast cell-laden bioink. The addition of nanofibers increased the ink’s viscosity and maintained a shear-thinning rheological response. The optimized concentration of nanofibers (1% *w*/*v*) allowed the printing of an accurate 3D grid structure of bioink. During the in vitro culture, the printed structure showed >80% cell viability and increased metabolic activity, indicating its potential for use in tissue engineering applications [49].

### 4.2. Other Polymeric-Nanofiber-Reinforced Ink

Teixeira MC et al. [50] developed lysozyme nanofiber (LNF)-reinforced alginate hydrogel bioinks. The LNFs were prepared by dissolving hen egg-white lysozyme in a buffer solution (10 mM HCl, pH 2) mixed with a solvent composed of choline chloride and acetic acid. The lyophilized nanofibers were suspended in alginate hydrogel at different concentrations (1, 5, and 10 wt%) and crosslinked using CaCl_2_ to formulate a printable ink. The integration of LNFs increased the mechanical properties, swelling and degradation, and morphology of the hydrogels. The printability (*Pr*) of the alginate-LNF inks was good, at approximately 0.9, due to the presence of the nanofibers. The printed HaCaT cell-laden bioink demonstrated a cell viability of 87.99 ± 1.28% for 7 days.

**Figure 7:**
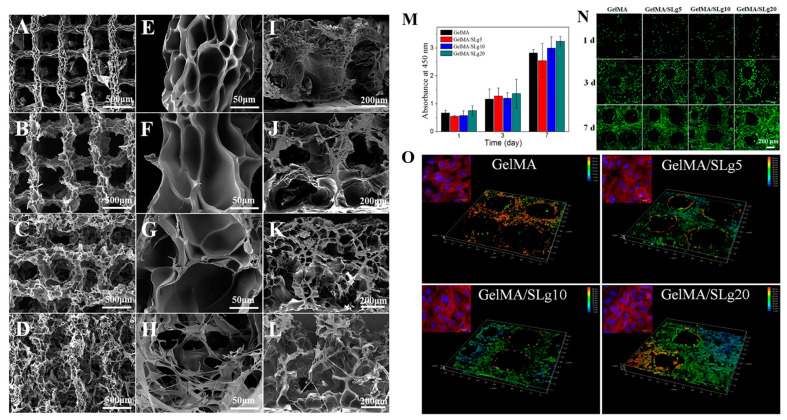
Three-dimensional-printed constructs composed of GelMA and self-assembling peptide nanofiber (SLg). SEM images of the printed scaffolds at different magnifications. (**A**,**E**,**I**) GelMA; (**B**,**F**,**J**) GelMA/SLg5; (**C**,**G**,**K**) GelMA/SLg10; and (**D**,**H**,**L**) GelMA/SLg20. Proliferation of L929 cells on different scaffolds. (**M**) CCK-8 assay findings on days 1, 3, and 7 (*n* = 3). (**N**) LIVE/DEAD fluorescent images of cells on scaffolds; (**O**) 3D reconstruction of cells grown at the corner of the scaffolds on day 7. Data reproduced from Ref. [51]. Copyright Elsevier 2021.

In a study, a proangiogenic self-assembling peptide nanofiber (SLg) was blended with GelMA to construct an interpenetrating polymer network, which exhibited improved elasticity and absorbency in 3D-printed scaffolds. SLg (CH_3_CO-SLSLSLSLSLSLKGEETEVTVEGLEPG-OH) was produced using Fmoc solid-phase chemistry. The in vitro biological studies showed that the scaffold provided an appropriate microenvironment for cellular migration and proliferation and supported the development of blood vessels. The in vivo studies uncovered that the scaffolds containing 20% peptide nanofibers demonstrated a collagenous fibrous structure, leading to enhanced revascularization and enabling dermal regeneration (Figure 7) [51].

**Figure 8:**
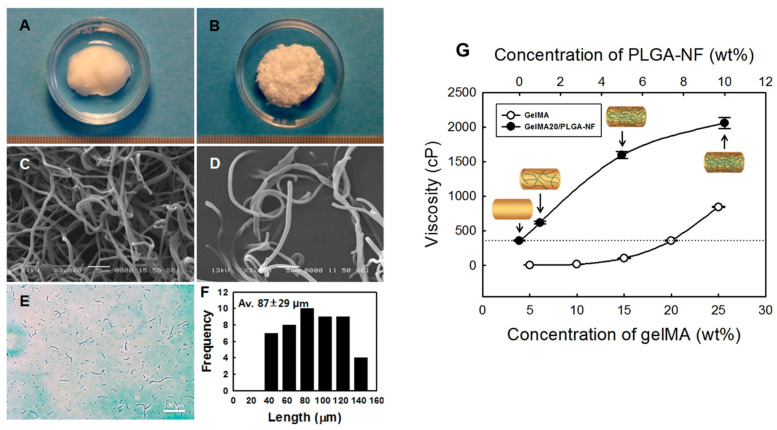
Digital images of the electrospun (**A**) PLGA nanofiber ball and (**B**) ground PLGA nanofiber fragments (PLGA-NF). Respective SEM images of (**C**) PLGA nanofiber balls and (**D**) PLGA-NF. (**E**) Optical microscope image of PLGA-NA. (**F**) Fiber length distribution (*n* = 50) of PLGA-NF. (**G**) Viscosity of gelMA and gelMA20/PLGA-NF solutions (*n* = 5). Data reproduced from Ref. [6]. Copyright Elsevier 2020.

A fibrous bioink composed of polylactic acid nanofibers, alginate, and human adipose-derived stem cells (hASCs) was bioprinted to fabricate musculoskeletal soft tissue constructs [52]. The PLA nanofibers were produced by infusing the polymer solution into a channel of viscous dispersion media composed of glycerin and deionized water in laminar Poiseuille shear flow, in which the polymer droplets were stretched into separate nanofibers. Initially, the cell-laden 3D-bioplotted strands were investigated for hASC viability and proliferation for 16 days. Then, a human medial knee meniscus model created by magnetic resonance images was printed for evaluation over 8 weeks. The results revealed that the nanofibrous bioink permitted a greater level of cell proliferation within printed strands, with the maximum on day 7 of the investigation, with 28.5% higher cell metabolic activity compared to that of the bioink without nanofibers. The histological findings revealed that the 3D-plotted meniscus construct allowed the cells to differentiate through the chondrogenic pathway and produce a matrix containing collagen and proteoglycan. In another similar work, poly(lactic-co-glycolic acid) (PLGA) nanofiber fragments were utilized to prepare fibrous gelMA hydrogel bioink to develop patient-specific soft tissue constructs (Figure 8). PLGA nanofiber fragments were prepared by homogenizing the PLGA electrospun nanofiber mat in an aqueous solution. It was reported that the addition of 1 wt% nanofiber fragments significantly increased the viscosity and compressive modulus of the resulting hydrogel. The bioprinted gelMA/PLGA nanofiber hydrogels demonstrated enhanced cell proliferation compared to the control groups. The nanofibrous fragments acted as an artificial ECM to augment cell attachment and cell spreading in the hybrid hydrogel, which was evidenced by the abundant cytoskeletons with F-actin filaments in the 15-day cell culture period [6].

Alginate-based nonsynthetic bioinks were prepared by incorporating silk nanofibrils (SNF) at different concentrations (1, 2, and 3 wt%). The extraction processes of SNF from silkworm cocoons were the removal of gum using sodium hydrogen carbonate solution, dissolution in LiBr solution, and probe sonication. The study highlighted that the addition of 2 wt% SNF to the printable ink notably increased its rheological and mechanical properties and cell viability. The nanofibrous materials in the hybrid hydrogel had a greater influence on increasing cell viability (by about 1.5 times) and cell proliferation (by about 5.6 times) compared to the control groups after 5 days of incubation [53]. Carbon nanofibers are the most promising nanofillers for developing electroconductive and printable scaffolds. The carbon nanomaterials within a gel matrix supply a large surface area for electron transfer [62]. In a study, the carbon-nanofiber-incorporated hydrogel constructs displayed remarkable mechanical performance, with a Young’s modulus of 534.7 ± 2.7 kPa and a conductivity of 4.1 × 10^−4^ ± 2 × 10^−5^ S/cm. These conductive hydrogels were suggested for cardiac or neuronal tissue engineering strategies as they enhanced cellular proliferation compared to controls [54].

## 5. Conclusions and Future Perspectives

Nanofiber-reinforced ink is an emerging approach in nanocomposite-reinforced printable inks to produce various printed structures with the desired size, shape, and mechanical properties, ensuring biocompatibility. We studied cellulose- and other polymeric nanofiber-material-incorporated printable inks that effectively enhance tissue regeneration in various kinds of tissue models, such as the skin, heart, and musculoskeletal soft tissues. The formulation of nanofiber-reinforced inks still requires more progress in the aspects of preparation and optimization of nanofiber materials through in-depth biological studies. Though the nature of polymeric components supports the viability of cells, the ability of cells to reside in the printed tissue structures without leaking into the medium depends on optimizing the concentration of the polymeric components and nanofiber additives. Our literature survey shows that printable polymeric-nanofiber-based inks, except for cellulose, have been less explored, though they have been widely investigated as hybrid hydrogels for TE applications. The reason might be that bioprinting using additive manufacturing is a recent, advanced technology in the biomedical field. Apart from in vitro biological studies, researchers should further carry out in vivo animal studies. The common post-crosslinking method, UV curing, triggers toxicity in the cells when exposure time is increased. Meanwhile, a shorter exposure time causes the leakage of cells. Nowadays, researchers are attempting to crosslink the gel using visible light instead of harmful UV light, which is a positive approach in bioprinting. The future of 3D bioprinting of nanofiber-reinforced inks is promising, leading to the promotion of advanced patient-specific tissues, organs, and devices and increasing their ease of commercialization.

## Figures and Tables

**Table 1:** In vitro TE applications of nanofiber-reinforced printable inks (bioink or biomaterial ink) and their specifications. Cellulose nanofiber (CNF), TEMPO-oxidized bacterial cellulose (TOBC), polylactic acid (PLA), poly(lactic-co-glycolic acid) (PLGA), primary rabbit fibrochondrocytes (rFCs), human adipose-derived stem cells (hASC). ↑ denotes enhanced/increased.

Nanofiber-Reinforced Printable Inks	Specifications	Crosslinking Agent	Type of Printable Ink and Cell Quantity	Biological Outcomes	TE Application
Quince seed mucilage/CNF	Grid structure, 5 layers, 30 mm × 30 mm, 25% infill density (or) disc-shaped structure, 25 mm, 50% infill density	CaCl_2_	Biomaterial ink HepG2, 50,000 cells/mL	↑ cell viability (≥90%), cell attachment, and proliferation	Soft tissues [46]
Gelatin/alginate/CNF	Square blocks (15 mm × 15 mm × 2 mm)	CaCl_2_	Bioink rFCs, 5 × 10^6^ cells/mL	↑ accumulation of collagen type I and type II	Meniscal reconstruction [21]
Sodium alginate/TOBC nanofiber	Beads	CaCl_2_	Bioink NIH3T3, 2.3 × 10^6^ cells/mL	↑ aggregation and proliferation of cells	Skin tissue [47]
Sodium alginate/laponite nanoclay/TOBC nanofiber	Grid structure, 10 mm × 10 mm × 1 mm, line spacing 0.8–1.2 mm (or) 20 mm × 20 mm × 3 mm, line spacing 2 mm	CaCl_2_	Biomaterial ink L929, 1 × 10^4^ cells/well	↑ cell-material interactions and cell spreading	Skin tissue [48]
Pectin/TEMPO-oxidized CNF	Printed rings (Ø internal = 2 cm, Ø external = 3.6 cm)	CaCl_2_	Bioink L929, 10 × 10^6^ cells/mL	↑ cell viability (≥80%) and metabolic activity	Skin tissue [49]
Alginate/lysozyme nanofiber	2 layers, 20 mm × 20 mm, line spacing 2.25 mm	CaCl_2_	Biomaterial ink HaCaT, 2 × 10^6^ cells/mL	↑ cell viability (>80%)	Skin tissue [50]
GelMA/peptide nanofiber	5 layers, fiber spacing 500 µm, layer height 150 µm	UV-curing	Biomaterial ink L929 and HUVECs, 1 × 10^5^ cells/mL	↑ formation of lumen structure and angiogenesis	Skin tissue [51]
Alginate/PLA nanofiber	Meniscus constructs, five strands, 25 mm × 0.5 mm × 0.5 mm, interstrand spacing 3 mm	CaCl_2_	Bioink hASC, 1.375 × 10^6^ cells/mL	↑ metabolic activity and cell proliferation	Musculoskeletal soft tissue [52]
GelMA/PLGA nanofiber	Rectangle-shaped construct,10 mm × 10 mm, thickness 5 mm	UV-curing	Bioink NIH3T3, 5.0 × 10^6^ cells/1.5 mL	↑ cell spreading and proliferation	Soft tissues [6]
Alginate/silk nanofibrils	Five-layer grid pattern, 1 × 1 cm^2^	CaCl_2_	Biomaterial ink L929, 1 × 10^4^ cells/well	↑ cell viability and proliferation	Soft tissues [53]
Alginate/gelatin/carbon nanofiber	2 layers, 2 mm per layer, 9:4 mm (w × h)	CaCl_2_	Biomaterial ink NIH3T3, 0.04 × 10^6^ cells/construct	↑ cellular attachment and proliferation	Myocardial and neuronal tissues [54]

## Data Availability

Not applicable.
